# Association of decreased expression of long non-coding RNA LOC285194 with chemoradiotherapy resistance and poor prognosis in esophageal squamous cell carcinoma

**DOI:** 10.1186/s12967-014-0233-y

**Published:** 2014-08-29

**Authors:** Yu-suo Tong, Xi-lei Zhou, Xiao-wei Wang, Qing-quan Wu, Tong-xin Yang, Jin Lv, Jin-song Yang, Bin Zhu, Xiu-feng Cao

**Affiliations:** Department of Surgical Oncology, Affiliated Nanjing Hospital of Nanjing Medical University and Oncology Center of Nanjing Medical University, Nanjing, Jiangsu China; Department of Medical Oncology, Affiliated Nanjing Hospital of Nanjing Medical University and Oncology Center of Nanjing Medical University, Nanjing, Jiangsu China; Department of Radiation Oncology, Affiliated Huai’an First Hospital of Nanjing Medical University, Huai’an, Jiangsu China; Department of Thoracic Surgery, Affiliated Huai’an First Hospital of Nanjing Medical University, Huai’an, Jiangsu China; Department of Medical Oncology, Affiliated Huai’an First Hospital of Nanjing Medical University, Huai’an, Jiangsu China

**Keywords:** Esophageal squamous cell carcinoma, Long non-coding RNA, LOC285194, Chemoradiotherapy, Prognosis

## Abstract

**Background:**

Expression of the long non-coding RNA (lncRNA) LOC285194 was previously shown to be correlated with aggressive clinicopathological features and poor prognosis in several cancers. The aim of the present study was to explore the relationship between LOC285194 expression and clinical outcomes in esophageal squamous cell carcinoma (ESCC), so as to assess whether it could be a novel biomarker for prognosis and prediction of response to therapy on ESCC patients.

**Methods:**

The method of quantitative real-time polymerase chain reaction (qRT-PCR) was used to measure LOC285194 expression in pretreatment biopsy specimens and matched normal tissue derived from ESCC patients who underwent preoperative chemoradiotherapy followed by surgical resection (CRT + S group; n = 55) or from those who received surgical resection alone (S group; n = 87). The association between LOC285194 expression and clinicopathological features and prognosis were then analyzed.

**Results:**

LOC285194 expression was significantly down-regulated in ESCC tumor tissues when compared with the adjacent normal tissues (*p <* 0.001). Low expression of LOC285194 was associated with larger tumor size *(p =* 0.002), advanced TNM stage (*p =* 0.018), more lymph node metastases (*p =* 0.013) and distant metastases (*p =* 0.015). In the CRT + S group, the pathological complete response rate was 57% (16/28) for the LOC285194-high group, and 15% (4/27) for the LOC285194-low group. Univariate analysis revealed that low expression of LOC285194 was significantly correlated with CRT response (*p =* 0.002). Moreover, Kaplan-Meier survival analysis revealed that patients with low expression of LOC285194 had a decreased disease free survival (DFS) (*p <* 0.001) and overall survival (OS) (*p <* 0.001). Multivariable analysis further identified low expression of LOC285194 as an independent prognosis factor for CRT response (*p =* 0.011), DFS (*p <* 0.001) and OS (*p =* 0.002).

**Conclusion:**

Decreased expression of LOC285194 could serve as a molecular marker to predict the clinical outcome of ESCC patients after surgery, and select patients who would benefit from preoperative CRT.

## Introduction

In Eastern Asia, 90% of the esophageal cancers are esophageal squamous cell carcinoma (ESCC) [1]. When treated exclusively by surgery, the prognosis of patients with ESCC is poor, with 5-year survival rates ranging from 9%–40% [2]. Although preoperative chemoradiotherapy (CRT) is commonly recommended by many physicians to improve the rate of curative resection and prolong survival, the clinical outcomes from randomized trials are equivocal [3-5]. Indeed, patients with the same pathological types and the same clinical stages show significantly different survival benefits after the identical treatment. It has been previously reported that patients with pathological complete response (pathCR) have a better clinical outcome than those with less than pathCR [6]. Therefore, the identification of molecular markers related to tumor response to CRT could help to select patients who are most likely to benefit from the preoperative CRT.

Long non-coding RNAs (lncRNAs) represent a new class of non- protein-coding RNAs which are longer than 200 bases, and do not function as templates for protein synthesis [7]. Previous studies have proved that lncRNAs play a critical role in the development and progression of cancer [8]. Additionally, multiple studies have indicated that the expression levels of lncRNAs are dysregulated in different kinds of tumors, including ESCC [9], and the aberrant expression of lncRNAs are correlated with metastasis, recurrence and prognosis. Specifically, the lncRNA HOTAIR has been shown to play a role in breast cancer, MALAT1 has been related to lung cancer, and HULC is reported to contribute to liver cancer [10-12]. Recently, the relatively new field of lncRNA research has focused on their value in the diagnosis and treatment of cancer. Indeed, it has been suggested that lncRNAs may serve as molecular markers for diagnosis, prognosis and prediction of tumor response to treatment [13].

The lncRNA LOC285194 (Gene ID: 285194), also called LSAMP antisense RNA 3, consists of four exons with 2105 bases in length. It was first reported to be within a tumor suppressor unit in osteosarcoma and depletion of this lncRNA promoted proliferation of normal osteoblasts through regulation of apoptotic and cell cycle transcripts as well as VEGF receptor 1 [14]. Decreased of LOC285194 expression has been reported in several cancers, and was strongly associated with malignant potential and poor patient prognosis [14-16]. However, to date, the association between this lncRNA expression and the prognosis of patients with ESCC has not been elucidated. In this study we examined the expression level of LOC285194 in ESCC tumors tissues and adjacent normal tissues. The relationships between its expression and clinicopathological features were then analyzed so as to evaluate whether LOC285194 expression could be a useful biomarker for prognosis and prediction of response to therapy in ESCC patients.

## Materials and methods

### Patients and samples

One hundred and forty-two tumor tissues and paired adjacent normal samples were obtained from patients who, based on the biopsy specimens, were diagnosed with ESCC at Nanjing Hospital Affiliated to Nanjing Medical University (n = 64) and Huai’an First Hospital Affiliated to Nanjing Medical University (n = 78) between January 2009 and November 2010. All of the specimens were immediately frozen in liquid nitrogen and then stored at –80°C until RNA extraction. Of the 142 patients with ESCC, 55 (39%) patients underwent surgical treatment four or five weeks after completion of preoperative CRT (CRT + surgery [CRT + S] group). The remaining 87 (61%) patients were administered with surgical resection alone (S group). All patients were selected in the study based on the following inclusion criteria: biopsy specimens were identified as ESCC by histopathological examination; no previous treatment had been received; no T4 or T1N0 tumors; and no evidences of distant organ metastasis. The clinical stage was determined according to the 7th edition of tumor-node-metastasis (TNM) classification for esophageal carcinoma (UICC, 2009). Written informed consent was obtained from all patients and the study was approved by the Ethics committee of Nanjing Hospital of Nanjing Medical University and Huai’an First Hospital of Nanjing Medical University.

### Treatment

CRT + S group: Each patient underwent the same preoperative concurrent CRT which included a 5-fluorouracil (5-FU) based regimen. Specifically, cisplatin (20 mg/m^2^/day, for 5 days, IV) and 5-FU (500 mg/m^2^/day, for 5 days, 24 h continuous infusion) were administered on days 1–5. Standard premedications were used. Radiotherapy was performed on the first day of the first chemotherapy cycle. A total radiation dose of 40 Gy was given in 20 fractions of 2 Gy each, with 5 fractions per week for 4 weeks. Four or five weeks after completion of CRT, a standard thansthoracic en bloc esophagectomy with two-field lymph node dissection was performed.

S group: The surgical approach was the same as that described for the CRT + S group.

### Evaluation and follow-up

The histopathological response to CRT was classified into four categories according to the following criteria: grade 1, no evidence of viable tumor cells; grade 2, <10% viable tumor cells; grade 3, 11–50% viable tumor cells; grade 4, >50% viable tumor cells [17]. We then divided the four categories into two groups: the pathological complete response (pathCR) group, which consisted of grade 1, and the < pathCR group, which consisted of grades 2–4.

All patients were followed every 3 months during the first year, every 6 months for an additional 2 years, and then every year until March 2014.

### Cell lines and cell culture

This study used five human ESCC cell lines (KYSE30, KYSE 70, KYSE 150, KYSE510 and Eca109) and one normal human esophageal epithelial cell (Het-1A). All the cells were generous gifts from Dr. Zhi-hua Liu at the State Key Laboratory of Molecular Oncology, Cancer Institute, Chinese Academy of Medical Sciences (Beijing, China) [9]. These cells were grown in RPMI-1640 (Invitrogen, Carlsbad, CA) medium supplemented with 10% fetal bovine serum (Gibco, Grand Island, NY) and 1% penicillin-streptomycin at 37°C with 5% CO_2_.

### RNA isolation and cDNA synthesis

Total RNAs were extracted from tumor tissues and adjacent normal tissues using Trizol reagent (Invitrogen, Carlsbad, CA, USA) according to the manufacturer’s instructions. The yield and quality of total RNA was evaluated by measuring the absorbance at 260 and 280 nm. Only samples with an A_260_:A_280_ ratio between1.8 and 2.1 was considered for further analysis. cDNAs were synthesized using the PrimeScript™ RT reagent kit with gDNA Eraser (Takara, Dalian, China) according to the manufacturer’s protocol. Briefly, 1 μg total RNA, 2 μl 5× gDNA Eraser Buffer, 1 μl gDNA Eraser and RNase Free dH_2_O, were combined in a total reaction volume of 10 μl and incubated at 42°C for 2 min to eliminate the genomic DNA. Ten microliters of reverse-transcription reaction mixture (consisted of 4 μl 5× PrimeScript Buffer 2, 1 μl PrimeScript RT Enzyme Mix 1, 1 μl RT Primer Mix, and 4 μl RNase Free dH_2_O) was then added, and the mixture was incubated at 37°C for 15 min, followed by 85°C for 5 s to generate the cDNA.

### Quantitative real-time PCR (qRT-PCR)

The expression of LOC285194 was quantified using SYBR® Premix Ex Tag™ II (Takara, Dalian, China) according to the manufacturer’s instructions on the ABI 7500 Real-Time PCR System (Applied Biosystems, Foster City, CA). Briefly, the 20 μl reaction mixtures were incubated at 95°C for 30 s for the initial denaturation stage, followed by 40 cycles at 95°C for 5 s and 60°C for 34 s. GAPDH was used to normalize the target gene expression. The ΔCt method was used to calculate the relative expression of LOC285194 in ESCC tumor tissues or ESCC cell lines in comparison with paired normal tissues or cells, respectively. Each sample was examined in triplicate. The primers used in this study were synthesized by Invitrogen with the sequences as follows: 5′- TGTGCCTGTTTGACCTCTGA-3′(sense) and 5′-AGGAAGGATAAA AGACCGACCA -3′(antisense) for LOC285194; 5′-TGCACCACCAACTGCTTAGC-3′(sense) and 5′-GGCATGGACTGTGGTCATGAG -3′ (antisense) for GAPDH.

### Statistical analysis

All statistical analyses were performed using the Statistical Program for Social Sciences (SPSS) 20.0 software. Overall survival (OS) was defined as the time from registration to death or until last follow-up date. Disease free survival (DFS) was calculated from registration to detection of tumor recurrence or until last follow-up date. The correlation between LOC285194 expression and clinicopathological characteristics were evaluated by the χ^2^ test. DFS and OS were calculated by Kaplan-Meier survival analysis and compared by the log rank test. To assess the factors that influenced the CRT response, multiple logistic regression analysis was performed on the factors that were shown to be significant on univariate analysis. All statistical analysis were two sided and *p* values less than 0.05 were considered significant.

## Results

### Patient characteristics

Patient clinicopathological characteristics were shown in Table 1. The cohort comprised 48 females and 94 males, with an average age of 64.5 years (range, 41 to 75). In the S group, clinical stages of the 87 patients were as follows: 13 cases, stage IIA; 21 cases, stage IIB; and 53 cases, stage III.

**Table 1:** **Correlation between LOC285194 expression and clinicopathologic characteristics of 142 ESCC patients**

**Variables**	**LncRNA LOC285194 subgroup** ^**a**^		***P*** ^**b**^ **value**
	**Low**	**High**	
**All cases**	**71**	**71**	
Gender			
Male	49 (69.1%)	45 (63.3%)	0.478
Female	22 (30.0%)	26 (36.7%)	
Age (years)			0.614
≤55	8 (11.3%)	10 (14.1%)	
>55	63 (88.7%)	61 (85.9%)	
Tumor size (cm)			0.002*
≤5	39 (55.0%)	57 (80.3%)	
>5	32 (45.0%)	14 (19.7%)	
Tumor location			0.573
Cervical/upper thoracic	6 (8.5%)	8 (11.3%)	
Middle/lower thoracic	65 (91.5%)	63 (88.7)	
Histologic grade			0.022*
G1	6 (8.5%)	7 (9.9%)	
G2	36 (50.7%)	50 (70.4%)	
G3/G4	29 (40.8%)	14 (19.7%)	
Smoking status			0.603
Ever/current	28 (39.4%)	25 (35.2%)	
Never	43 (60.6%)	46 (64.8%)	
Alcohol consumption			0.206
Ever/current	17 (23.9%)	11 (15.5%)	
Never	54 (76.1%)	60 (84.5%)	
T status			0.27
T1/2	18 (25.4%)	24 (33.8%)	
T3/4	53 (74.6%)	47 (66.2%)	
N status			0.013*
N0M0	23 (32.4%)	39 (54.9%)	
N1M0	22 (31.0%)	19 (26.8%)	
M1-lym^c^	26 (36.6%)	13 (18.3%)	
M status			0.015*
M0	45 (63.4%)	58 (81.7%)	
M1-lym	26 (36.6%)	13 (18.3%)	
Clinical stage			0.018*
IandII	26 (36.6%)	40 (56.3%)	
III	45 (63.4%)	31 (43.7%)	
CRT response	27	28	0.001*
PathCR^d^	4 (29.6%)	16 (67.9%)	
Less than PathCR	23 (70.4%)	12 (32.1%)	

^a^Median expression level was used as a cutoff to divide the 142 patients into LOC285194 low group (n = 71) and LOC285194 high group (n = 71). ^b^Chi-square test. ^c^M1-lym, distant lymph node metastasis. ^d^PathCR, pathologic complete response. *p < 0.05.

In the CRT + S group, all 55 patients underwent the same preoperative concurrent CRT regimen. Twenty patients achieved pathCR, and the less than pathCR was observed in the remaining 35 patients.

Of the 142 patients with ESCC, the median follow-up time was 23 months (range, 3 – 36 months), with 97 (68%) tumor recurrence and 89 (63%) cancer-related death.

### Expression of LOC285194 in ESCC

**Figure 1:**
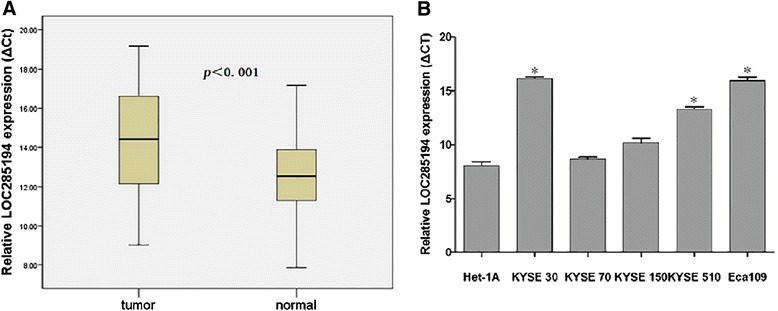
**qRT-PCR analysis of lncRNA LOC285194 expression in ESCC. (A)** LOC285194 was significantly down-regulated in 142 ESCC tissues, compared to the adjacent normal tissues. Statistical differences were analyzed using paired *t* test. ΔCt method was used to measure the relative LOC285194 expression, which was normalized by the GAPDH expression level. **(B)** LOC285194 expression was evaluated by qRT-PCR in 5 ESCC cell lines. Normal human esophageal epithelial cell was used as control. *p < 0.05. Smaller ΔCt value indicates higher expression.

To assess whether LOC285194 expression was associated with clinicopathologic characteristics in ESCC, we first measured its expression level by qRT-PCR in 142 paired tumor samples and adjacent non-tumor tissues. We detected that LOC285194 expression was significantly down-regulated in tumor tissues when compared to the adjacent normal tissues (*p <* 0.001; Figure 1A). Approximately 69% (98/142) of the tumor samples had >2-folds lower LOC285194 expression than that in non-tumor tissues. Furthermore, we also examined the level of LOC285194 in five ESCC cell lines and one normal esophageal epithelial cell. Among the 5 tumor cell lines, the LOC285194 level was lower in 3(KYSE30, KYSE510, Eca109) as compared with the normal esophageal epithelial cell (*p <* 0.05; Figure 1B), but no statistical differences were observed for KYSE70 and KYSE150.

### LOC285194 expression and clinicopathological characteristics

To further analyze whether there was an association between LOC285194 expression and specific clinicopathological parameters in patients with ESCC, we then used the median expression level of LOC285194 as a cutoff to divide the 142 patients into LOC285194-high (n = 71, with an average ∆Ct expression value of 8.29) and low groups (n = 71, with an average ∆Ct expression value of 11.96). We found that the LOC285194-low group was significantly associated with larger tumor size, worse histologic grade, advanced TNM stage, and more lymph node metastasis and distant metastases (p < 0.05; Table 1). However, statistical association between LOC285194 expression and other clinicopathological data including gender, age, tumor location, smoking status, alcohol consumption and depth of invasion released no significant differences (p > 0.05; Table 1).

### Correlation between LOC285194 expression and pathological response to CRT

We next sought to explore whether LOC285194 expression could be a predictable molecular marker for CRT response on ESCC patients. We first investigated the association between LOC285194 expression level and pathological response to CRT in the CRT + S group. Of the 55 patients, the pathCR rate was 57% (16/28) in the LOC285194- high group, while it was only 15% (4/27) in the LOC285194-low group. The LOC285194-low group had a significantly lower response to CRT than that of LOC285194-high group(*p =* 0.001). In other words, patients with low expression of LOC285194 showed resistance to CRT. These data suggested that LOC285194 could be a useful molecular maker for predicting the sensitivity to CRT on ESCC patients. Consequently, univariate analysis was then performed to determine whether LOC285194 was a feasible molecular maker for prediction of response to CRT, the results revealed that only LOC285194 expression was associated with tumor response to CRT (OR, 0.130; 95% CI, 0.036–0.478; *p =* 0.002; Table 2). Unexpectedly, no other clinical factors were observed to be associated with CRT response. However, age, gender, tumor size, tumor location, lymphatic metastases, distant metastases and clinical stage trended toward a correlation with CRT response and were included in further multiple logistic regression analysis along with LOC285194. Multivariate analysis indicated that low expression of LOC285194 was the only independent risk factor for CRT response (OR, 0.133; 95% CI, 0.028–0.624; *p =* 0.011, Table 2).

**Table 2:** **Univariate and multivariate logistic regression analysis of factors associated with pathologic response**

				**Univariate analysis**			**Multivariate analysis**		
**Variables**	**Case**	**PathCR**	**<pathCR**	**OR**	**95% CI**	***p*** **value**	**OR**	**95% CI**	***p*** **value**
Ages									
≤55	12	2	10	0.278	0.054-1.424	0.125	0.302	0.049-1.874	0.199
>55	43	18	25						
Gender									
Male	34	9	25	0.372	0.104-1.030	0.056	0.415	0.103-1.676	0.217
Female	21	11	10						
Tumor size (cm)									
≤5	35	14	21	1.556	0.482-5.019	0.460	0.617	0.131-2.905	0.541
>5	20	6	14						
Tumor location									
Cervical/upper thoracic	14	7	7	2.154	0.625-7.421	0.224	1.757	0.372-8.298	0.477
Middle/lower thoracic	41	13	28						
Histologic grade									
G1	8	3	5	0.873	0.172-4.429	0.870	nd	nd	nd
G2	27	11	16	1.400	0.250-7.830	0.702	nd	nd	nd
G3	20	6	14						
T status									
T1/2	14	6	8	1.446	0.419-4.997	0.560	nd	nd	nd
T3	41	14	27						
N status									
Negative	20	10	10	2.500	0.797-7.839	0.116	0.508	0.027-9.577	0.651
Positive	35	10	25						
M status									
M0	39	16	23	2.087	0.586-7.651	0.267	1.250	0.212-7.368	0.805
M1-lym	16	4	12						
Clinical stage									
IandII	21	10	11	2.182	0.705-6.756	0.176	2.102	0.109-40.483	0.623
III	34	10	24						
LOC285194									
low	27	4	23	0.130	0.036-0.478	0.002*	0.133	0.028-0.624	0.011*
high	28	16	12						

PathCR, pathologic complete response; OR, odd ratio; CI, confidence interval; M1-lym, distant lymph node metastasis; nd, not done. *p < 0.05.

### Association between LOC285194 expression and prognosis after esophagectomy

**Figure 2:**
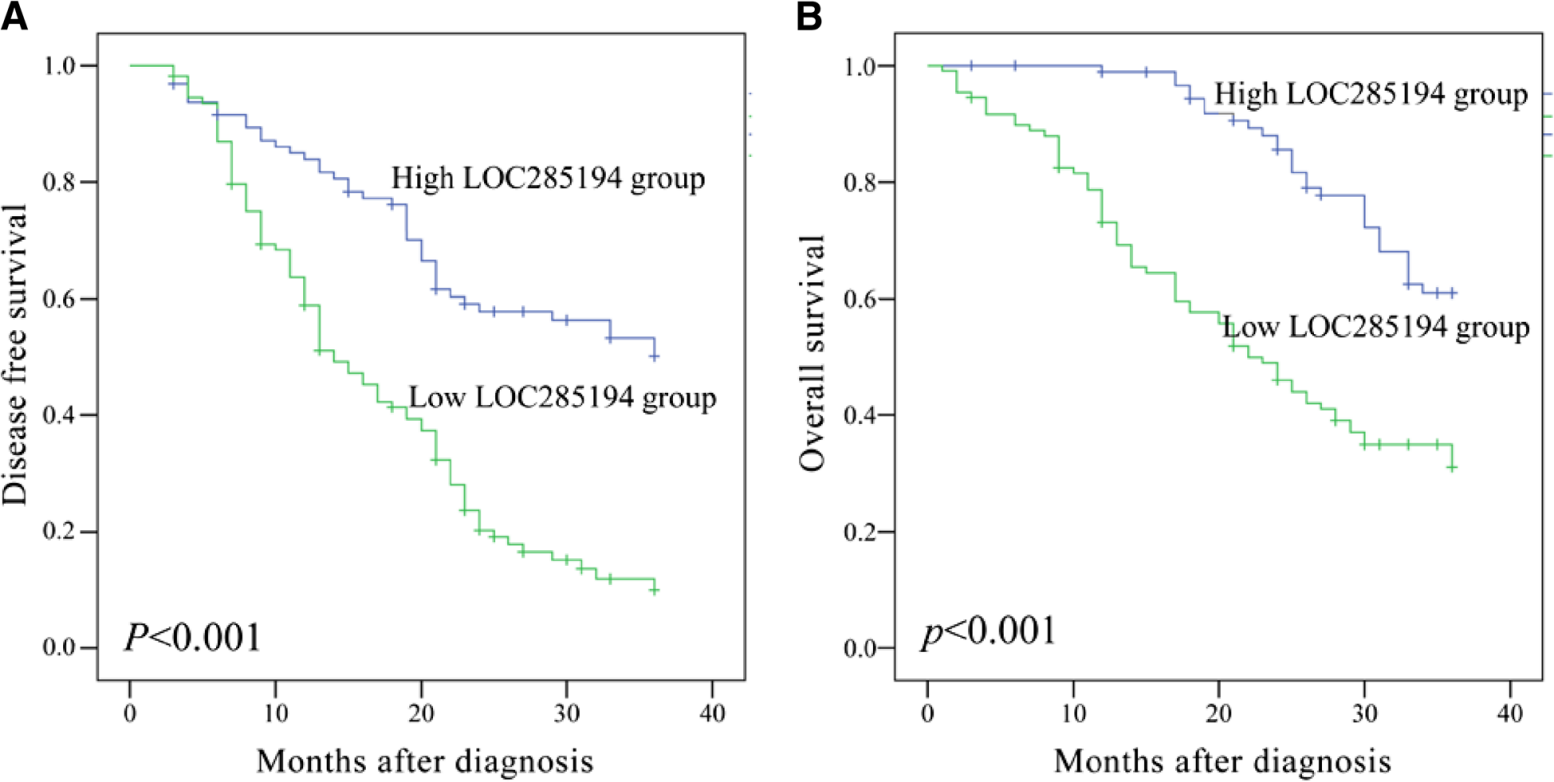
**Kaplan-Meier survival analysis of association between LOC285194 expression level and (A) DFS and (B) OS of 142 ESCC patients.** Patients with low expression of LOC285194 showed decreased DFS (p < 0.001) and OS (p < 0.001 ) compared with patients with high level of LOC285194 expression. Survival curves were compared using log-rank test.

Kaplan-Meier survival analysis was used to investigate the association between the LOC285194 expression and the prognosis of patients with ESCC after esophagectomy. From the Kaplan-Meier survival curves, we found that patients with low expression of LOC285194 had poorer DFS (*p <* 0.001; Figure 2A) and OS (*p <* 0.001; Figure 2B) as compared with the LOC285194-high group. Moreover, multivariate analysis revealed that low expression of LOC285194 (HR, 0.341; 95% CI, 0.193–0.602; *p <* 0.001 and HR, 0.388; 95% CI, 0.210–0.715; *p =* 0.002 for DFS and OS, respectively) as well as distant metastasis (HR, 2.123; 95% CI, 1.063–4.240; *p =* 0.033 and HR, 2.389; 95% CI, 1.132–5.041; *p =* 0.022 for DFS and OS, respectively) were independent prognosis factors that affected the DFS and OS of ESCC patients after esophagectomy (Table 3).

**Table 3:** **Multivariate cox regression analysis of independent predicting factors for disease free survival and overall survival**

	**Disease free survival**			**Overall survival**		
**Factors**	**HR**	**95% CI**	***P*** **value**	**HR**	**95% CI**	***P*** **value**
Tumor size (>5:≤5)	1.137	0.650-1.991	0.653	1.030	0.564-1.880	0.924
WHO grade (G3,G4/G1,G2)	1.019	0.607-1.981	0.760	1.046	0.553-1.979	0.889
T status (T3/T31,T2)	1.503	0.675-3.347	0.318	0.773	0.299-1.997	0.594
N status (positive/negative)	1.360	0.539-3.431	0.514	1.360	0.467-3.996	0.573
M status (M1-lym/M0)	2.123	1.063-4.240	0.033*	2.389	1.132-5.041	0.022*
Clinical stage (III/I + II)	1.179	0.427-3.256	0.750	2.408	0.735-7.882	0.146
LOC285194 (low/high)	0.341	0.193-0.602	0.001*	0.388	0.210-0.715	0.002*

HR, hazard ratio; CI, confidence interval; M1-lym, distant lymph node metastasis; *P < 0.05

## Discussion

Esophageal squamous cell carcinoma (ESCC) is one of the most virulent malignancies worldwide with the 5-year survival rate less than 30% [18]. Although there have been recent improvements in combination treatments including chemoradiotherapy(CRT) alone or as an adjunct prior to surgery, local recurrences and distant metastases are still common, and the median survival time for ESCC patients continues to be poor. The dismal outcomes of ESCC are largely due to our inability to select the optimum therapy and CRT resistance [3]. Therefore, the exploration of novel molecular markers is very important to help select patients who will benefit from CRT as well as identify therapeutic targets.

LncRNAs are a new class of transcripts recently discovered to be pervasively transcribed in human genome and play a critical role in epigenetic regulation [19]. In addition, as with microRNA, lncRNAs may function as tumor markers for the prediction of tumor prognosis [13,20]. Here we reported lncRNA LOC285194, which was previously shown to function as a tumor suppressor in osteosarcoma and was significantly down-regulated in 43 osteosarcoma tumor samples and 5 osteosarcoma cell lines [14]. In addition, expression of LOC285194 deletion in tumor was found to be associated with poor prognosis of osteosarcoma patients. The misexpression of this lncRNA was also discovered in colorectal cancer, and patients with low expression of LOC285194 had a shorter DFS [16]. This common characteristic thus strengthened the clinical application value of LOC285194. Therefore, we hypothesized that LOC285194 expression was also decreased in ESCC tumor tissues and decreased of this lncRNA may influence the treatment outcomes and could predict the prognosis of ESCC patients.

To test this hypothesis, tissue samples from 142 patients with ESCC were selected. The qRT-PCR showed that LOC285194 was significantly down-regulated in ESCC tumor tissues compared to the adjacent nontumor tissues. Furthermore, this significantly lower expression was also discovered in 3 ESCC cell lines when compared with that in normal esophageal epithelial cell. We then used the median expression level of LOC285194 as a cutoff to divide the 142 patients into the LOC285194-low group and the LOC285194-high group to further investigate the association between LOC285194 expression and clinicopathological characteristics. Patients with low expression of LOC285194 showed larger tumor size, worse histologic grade, advanced TNM stage, more lymph node metastasis and distant metastases than the LOC285194-high group. Namely, the low expression level of LOC285194 was associated with malignant potential and aggressive clinicopathological features in ESCC. However, the exact mechanisms behind the dysregulation of LOC285194 expression in ESCC remain unclear. In the study by Liu Q et al, LOC285194 was found to be specifically induced by wild-type p53, p53 directly interacted with the p53 response element in the upstream of LOC285194 to induce its transcription [15]. Decreased expression of LOC285194 could be explained by p53 status, because p53 mutation or deletion could account for more than 50% of ESCC cases[21-23].

In patients with ESCC, preoperative CRT is often performed as an adjuvant treatment to improve survival [24]. However, only 13–49% patients who have pathCR will benefit from the treatment, the remaining 50% patients present the CRT resistance[25]. To our knowledge to date, no studies have been performed regarding the association between lncRNA expression and the pathologic response in ESCC patients receiving preoperative CRT. Here, we demonstrated for the first time that low expression of LOC285194 was significantly negatively correlated with pathologic response to CRT. In other words, patients with low expression of LOC285194 suggested resistance to CRT. Indeed, in the CRT + S group, we found that 57% (16/28) of the LOC285194- high group had a pathCR to CRT compared with only 15% (4/27) in the LOC285194-low group. Therefore, our results indicated that low expression of LOC285194 could be a valuable molecular marker for personal treatment screening of ESCC patients before esophagectomy. In addition, rescuing the expression of LOC285194 may have a novel clinical application in the treatment of ESCC patients. However, the exact mechanisms underlying how misexpression of LOC285194 influences the CRT sensitivity have not been fully understood. It has been reported that LOC285194 is located at osteo3q13.31, which harbors frequent focal copy number alterations (CNAs) and loss of heterozygosity (LOH) in osteosarcoma. And depletion of LOC285194 promotes osteoblast proliferation *in vitro* through regulation of cell cycle transcripts such as cyclin D1 and VEGF/VEGFR1 [14]. Interestingly, focal osteo3q13.31 depletion and LOH are also found in various tumors including esophageal cancer [26]. In addition, previous reports have shown that up-regulation of cyclin D1, VEGF and VEGFR1 are associated with CRT resistance and poor prognosis in ESCC patients [27,28]. Thus, we hypothesize that changes LOC285194 might be involved in the regulation of the expression of proliferation-associated genes that in turn may partly contribute to tumor resistance in preoperative CRT. Clearly, further cell experiments are needed to fully understand the oncogenic function of LOC285194 in human ESCC pathogenesis, including the elucidation of which signaling pathways are involved in resistance to CRT.

One of the most important findings in the present study was the prognostic significance of LOC285194 expression in ESCC. We observed a close association between low expression of LOC285194 and shorter DFS and OS in patients with ESCC. Furthermore, the multivariate analysis showed that low expression of LOC285194 was a powerful independent prognosis factor of poor DFS and OS, which was consistent with the previous reports in colorectal cancer and osteosarcoma. These findings suggested that low expression of LOC285194 could signify a higher risk of disease recurrence and/or treatment failure, and, that postoperative ESCC patients should be closely monitored and receive effective adjuvant therapies.

## Conclusion

Our study shown that LOC285194 expression was significantly down-regulated in ESCC tumor tissues and cell lines, and low expression of LOC285194 was associated with CRT resistance and poor prognosis. Furthermore, decreased expression of LOC285194 could potentially serve as a molecular marker to predict the clinical outcomes (shorter DFS and OS) of ESCC patients after surgery, and select patients who will benefit from the preoperative CRT.
